# Age‐ and sex‐related changes in rat renal function and pathology following neonatal hyperoxia exposure

**DOI:** 10.14814/phy2.12887

**Published:** 2016-08-15

**Authors:** Megan R. Sutherland, Chanel Béland, Marie‐Amélie Lukaszewski, Anik Cloutier, Mariane Bertagnolli, Anne Monique Nuyt

**Affiliations:** ^1^ Sainte‐Justine University Hospital and Research CenterMontrealQuebecCanada; ^2^ Department of PediatricsUniversité de MontréalMontrealQuebecCanada

**Keywords:** Hyperoxia, nephrogenesis, preterm birth, renal disease

## Abstract

Preterm neonates are prematurely exposed to high oxygen levels at birth which may adversely impact ongoing renal development. The aim of this study was to determine the effects of neonatal hyperoxia exposure on renal function and morphology with aging. Sprague Dawley rat pups were raised in a hyperoxic environment (80% oxygen) from P3 to P10 during ongoing postnatal nephrogenesis. Control litters were kept in room air (*n* = 6–8 litters/group; one male, one female/litter/age). Kidney function (urine and plasma creatinine, sodium, and protein) and morphology (renal corpuscle size, glomerulosclerosis, fibrosis, and glomerular crescents) were assessed at 1, 5, and 11 months of age. Neonatal hyperoxia exposure had no impact on body or kidney weights. Creatinine clearance was significantly reduced following hyperoxia exposure at 5 months; there was no significant effect on renal function at 1 or 11 months. The percentage of crescentic glomeruli (indicative of glomerular injury) was markedly increased in 11 month hyperoxia‐exposed males. Renal corpuscle size, glomerulosclerosis index, and renal fibrosis were not affected. Findings suggest that exposure to high oxygen levels during development may impact renal functional capacity and increase susceptibility to renal disease in adulthood depending on age and sex.

## Introduction

There is emerging evidence linking preterm birth (birth prior to 37 completed weeks of gestation) with impaired renal development (Rodriguez et al. 2004; Sutherland et al. 2011), reduced kidney size (Keijzer‐Veen et al. 2010; Zaffanello et al. 2010; Kwinta et al. 2011), renal dysfunction (Rodriguez‐Soriano et al. 2005; Iacobelli et al. 2007), hypertension (Dalziel et al. 2007; Cooper et al. 2009; de Jong et al. 2012), and renal disease (Hodgin et al. 2009; Ikezumi et al. 2013). Exposure to high oxygen levels in the early postnatal period, when nephrogenesis is still ongoing, has been proposed to be one factor which may impair development and/or injure the immature kidneys of preterm neonates (Gubhaju et al. 2011; Sutherland et al. 2014). This may occur through either exposure to room air or mechanical ventilation (up to 100% supplemental oxygen, Tan et al. 2005; Rabi et al. 2011); blood oxygen concentrations rise immediately after birth (Kamlin et al. 2006; Rabi et al. 2006) compared to in utero which is an optimal (low oxygen: Rodesch et al. 1992; Fischer and Bavister 1993) environment for fetal kidney development (Tufro‐McReddie et al. 1997). These high oxygen levels cause oxidative stress, with preterm neonates particularly vulnerable due to their low antioxidant activity (Georgeson et al. 2002; Lee and Chou 2005). Oxidative stress is implicated in a number of diseases of prematurity, such as bronchopulmonary dysplasia and retinopathy, which are characterized by impaired angiogenesis, apoptosis, and inflammation (Smith 2003; Thebaud and Abman 2007; Saugstad et al. 2012).

We have previously shown in a rodent model of neonatal hyperoxia exposure (80% O_2_ from postnatal days [P] 3 to 10, encompassing a period of active postnatal nephrogenesis in the rat) that there are indications of impaired renal development, with pups exhibiting a significantly reduced nephrogenic zone and glomerular size at P5 (Popescu et al. 2013). At P10 there was no significant impact on nephron number. In adulthood, however, hyperoxia‐exposed animals exhibited a 25% reduction in nephron number, and also high blood pressure from ~8 weeks of age (Yzydorczyk et al. 2008; Bertagnolli et al. 2014). Therefore, we hypothesized that neonatal hyperoxia exposure would lead to renal dysfunction and renal pathology, and that these findings would worsen with age. The aim of this study was to comprehensively examine renal function and pathology in aging rats that were exposed to hyperoxia as neonates.

## Methods

### Animals

All studies were approved by the Animal Care Committee of the CHU Sainte‐Justine, and the treatment and care of animals was in accordance with the Guide for the Care and Use of Experimental Animals from the Canadian Council on Animal Care.

Sprague Dawley rat pups (Charles River, St.‐Constant, Québec, Canada) were continuously exposed to 80% O_2_ (mixture of medical grade 100% O_2_ and room air; Oxycycler ProOx model 110; Biosherix, Lacona, NY) from the third (P3) to the tenth (P10) day of life (hyperoxia group: H) (Yzydorczyk et al. 2008; Popescu et al. 2013). To avoid O_2_ toxicity, the mother of the O_2_‐exposed litter was interchanged every 12 h with another dam in room air. The pups of the dam used for interchange served as a control for the effects of the dam being exposed to hyperoxia, but the pups themselves were maintained in room air (normoxia–hyperoxia group: NH) (Popescu et al. 2013). We have previously shown in this model that blood pressure and nephron number in adulthood are not affected by dam interchange per se (Yzydorczyk et al. 2008). Control litters were kept with their own dams in room air (normoxia group: control). Given the timing of nephrogenesis, the commencement of oxygen exposure in the rats at P3 may be comparable to extremely preterm infants at ~24–25 weeks gestation. It is difficult to make a direct correlation between rats and humans, however, due to significant differences in the temporal and spatial development of the kidneys between species (Cullen‐McEwen et al. 2015).

All litters were equalized to *n* = 12 at P3, and pups were weaned at 4 weeks of age. At weaning, one male and one female pup was chosen at random for analysis at each time point: 1 (at weaning), 5 (early adulthood), and 11 (mid‐adulthood) months of age (*n* = 6–8 animals per sex/group/age). Animals were weighed weekly until 4 months, then monthly from 5 to 11 months.

### Renal function analyses

At 1, 5, and 11 months of age, male and female rats were placed individually into metabolic chambers overnight for 16 h to collect urine. At the conclusion, animals were anesthetized with isofluorane gas, and arterial blood was collected via cardiac puncture into heparinized tubes (prior to necropsy). Plasma was then separated following centrifugation. All urine and plasma samples were stored at −80°C until analysis.

Samples were assessed by the CHU Sainte‐Justine Biochemistry laboratory (Architect c8000, Abbott, Abbott Park, IL). Urine and plasma creatinine was measured by the Jaffe reaction. Creatinine clearance (mL/min/kg) was calculated using the formula: (Urine creatinine [mmol/L]/Plasma creatinine [mmol/L]) × (Urine volume/Time [mL/min])/Body weight (kg). Urine and plasma sodium was measured by an indirect ion‐selective electrode method. The fractional excretion of sodium (FENa, %) was calculated as: (Urine sodium [mmol/L]/Plasma sodium [mmol/L]) × (Plasma creatinine [mmol/L]/Urine creatinine [mmol/L]) × 100. Urine total protein was quantified by turbidimetric assay (benzethonium chloride) and urine albumin by immunoturbidimetry; levels were corrected by urine creatinine.

### Kidney tissue collection and processing

Under isofluorane gas anesthesia, animals were euthanized by decapitation. The left kidney from each animal was collected and immersion‐fixed in 10% buffered formalin. Fixed kidneys were cleaned of connective tissue, weighed, and sliced into quarters. Two opposing quarters were embedded in paraffin, and 4 μm sections along the coronal plane were stained with periodic acid Schiff (PAS) and picrosirius red. For all kidney analyses, researchers were blinded to the experimental group assignment.

### Assessment of renal corpuscle size, glomerulosclerosis, and glomerular crescents

PAS‐stained sections (one per animal) underwent systematic random sampling at a step length of 2 mm. At each field of view, photomicrographs were taken using a 20× lens. Image analysis software (ImageJ 1.36b, http://rsbweb.nih.gov/ij) was utilized to trace the boundary of the Bowman's capsule of each complete renal corpuscle and average renal corpuscle cross‐sectional area per kidney was then determined (Sutherland et al. 2011). The extent of glomerulosclerosis in each glomerulus was also categorized (Sutherland et al. 2013; Black et al. 2015) – Stage 1: 1–25%, Stage 2: 26–50%, Stage 3: 51–75%, and Stage 4: ≥76%. The glomerulosclerosis index for each kidney was calculated as: (1 × *n* Stage 1) + (2 × *n* Stage 2) + (3 × *n* Stage 3) + (4 × *n* Stage 4)/Total *n* of glomeruli (Maric et al. 2004). In these same images, the number of glomeruli exhibiting glomerular crescents, or not, was recorded. Crescents were defined as an accumulation of extracellular material and/or cells in the Bowman's space (Cohen 2006), with no distinction made as to the composition or size of the crescents. The percentage of crescentic glomeruli per kidney was then determined.

### Assessment of renal fibrosis

In picrosirius red‐stained sections (one per kidney), six images were taken at random of the renal cortex. ImageJ was utilized to determine the area of collagen (red) staining, and the average fibrosis per kidney was then determined. Glomeruli and interstitium were analyzed separately, and any areas of perivascular fibrosis were excluded (to prevent overestimation of interstitial fibrosis).

### Assessment of macrophage number

Macrophage number was examined in this study given the known association between oxidative stress and inflammation (Salzano et al. 2014; Sutherland et al. 2014), as well as macrophage number being a strong correlate of both renal interstitial and glomerular disease progression (Duffield 2010). The number of CD68+ macrophages was assessed using immunohistochemistry. Dewaxed sections underwent heat‐induced antigen retrieval in Tris‐Ethylenediaminetetraacetic acid (EDTA) buffer (10 mmol/L Tris Base, 1 mmol/L EDTA, 0.05% Tween‐20, pH 9.0), were blocked with H_2_O_2_ followed by goat serum, and then incubated overnight at room temperature with mouse anti‐CD68 (Ab31630, Abcam, Toronto, ON, Canada) at 1:200 dilution. Sections were then incubated with a horseradish peroxidase (HRP)‐conjugated goat anti‐mouse secondary antibody (Santa‐Cruz, Dallas, TX) at 1:400 for 90 min. 3,3'‐diaminobenzidine (DAB) was used to visualize antibody binding, and sections were counterstained with hematoxylin. Sections with the primary antibody excluded served as negative controls. Systematic random sampling at a step length of 2 mm was performed, with all positively stained cells (glomerular and interstitial) recorded at each field of view. Any cells within vessel lumens were excluded. The average number of CD68+ cells per field of view was calculated for each kidney.

### Statistical analysis

Data were analyzed using GraphPad Prism v.5 for Windows (GraphPad Software, San Diego, CA), and are presented as the mean ± SEM. Differences between the control, NH, and H groups, and age (1, 5, and 11 months) were analyzed using a two‐way analysis of variance (ANOVA) with the factors group (*P*_G_), age (*P*_A_), and their interaction (*P*_G × A_). Differences between males and females, and groups, were assessed at each time point using two‐way ANOVA, with the factors group (*P*_G_), sex (*P*_S_), and their interaction (*P*_G × S_). Body weights were analyzed using two‐way ANOVA with repeated measures. To determine differences between individual groups, each two‐way ANOVA was followed by a Bonferroni post hoc test. Statistical significance was accepted at the level of *P *<* *0.05.

## Results

### Body and kidney weights

**Figure 1:**
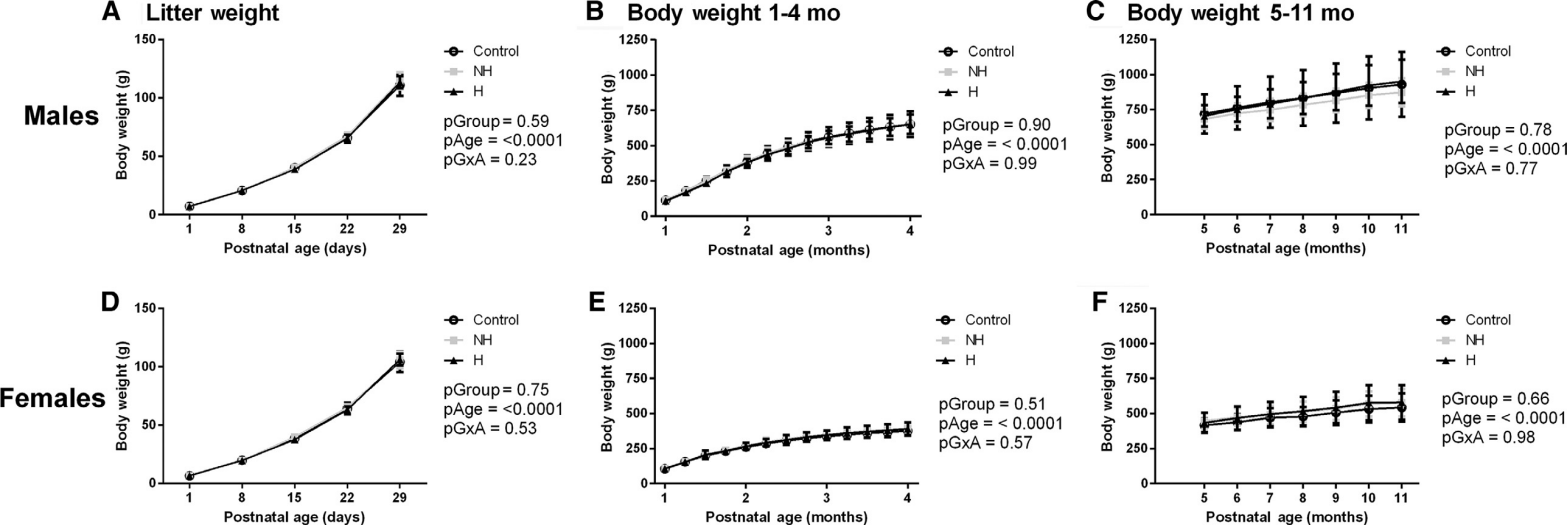
Body weights. Average litter weight before weaning (A, males; D, females), body weight from 1 to 4 months of age (B, males; E, females), and body weight from 5 to 11 months of age (C, males; F, females) in control, NH, and H rats. Analysis by two‐way ANOVA, with the factors group, age, and their interaction (*P*_G × A_). NH, normoxia–hyperoxia; ANOVA, analysis of variance.

**Figure 2:**
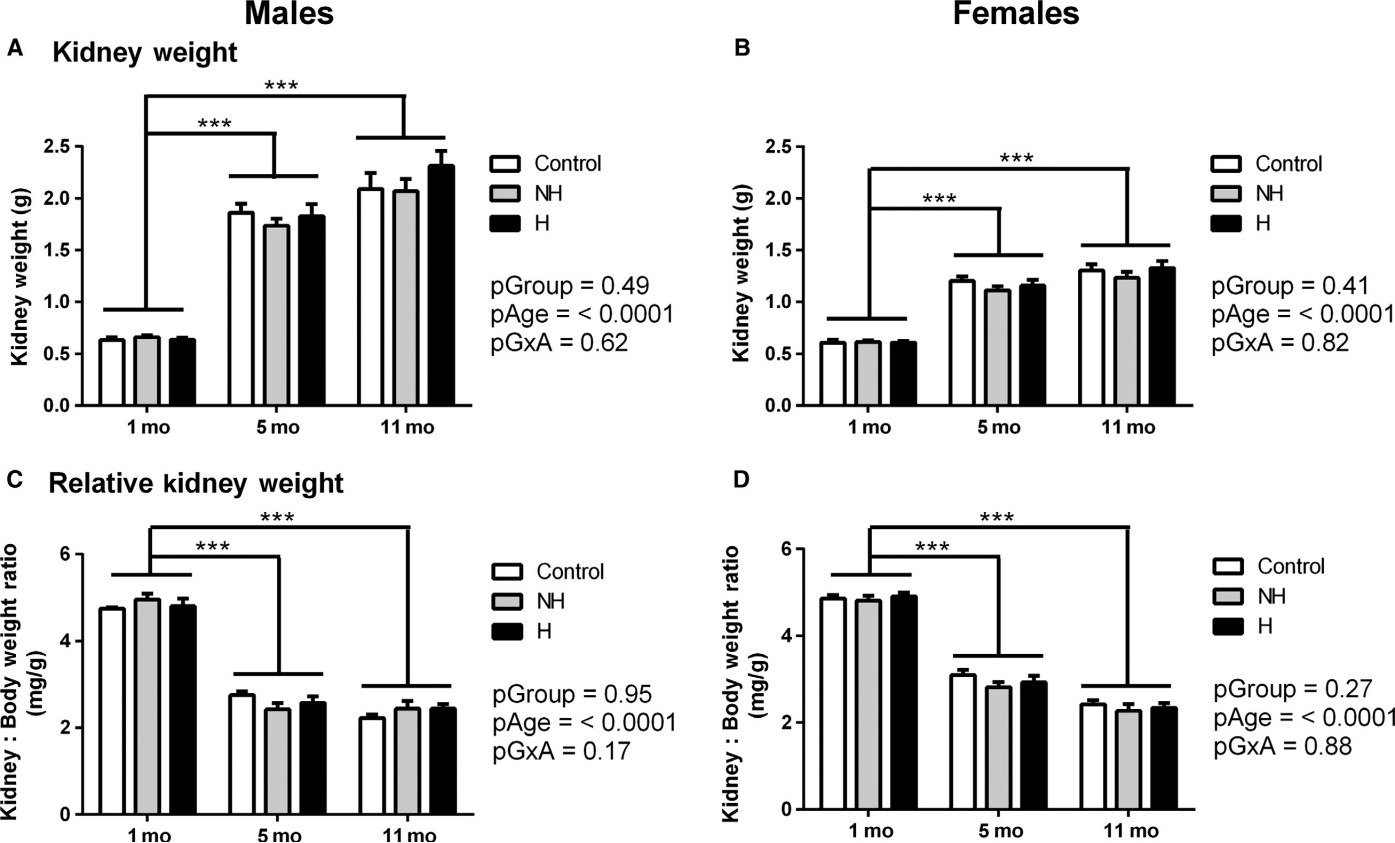
Kidney weights. Kidney weight (A, males; B, females) and kidney weight to body weight ratio (C, males; D, females) in rats at 1, 5, and 11 months of age. Analysis by two‐way analysis of variance (ANOVA), with the factors group, age, and their interaction (*P*_G × A_). ***P <* *0.0001 between groups as indicated, according to Bonferroni post hoc analysis.

Males were significantly larger, and had significantly larger kidneys, compared to females at 5 (*P* < 0.0001) and 11 (*P* < 0.0001) months of age, but not at 1 month (*P* = 0.06). There was no difference in body weight between groups at any age, in either males (Fig. 1A–C) or females (Fig. 1D–F). Absolute kidney weights significantly increased and relative kidney weights significantly decreased with increasing age, in both males (Fig. 2A and C) and females (Fig. 2B and D). There was no impact of hyperoxia exposure on kidney weight.

### Renal function

At 1 month of age, there were no differences between groups or sexes in any of the measured renal function parameters. At 5 months of age, in comparison to males the females exhibited significantly lower creatinine clearance (*P* < 0.0001) and urine total protein (*P* < 0.0001), and greater plasma creatinine (*P* = 0.02) and FENa (*P* < 0.0001). Importantly, at 5 months of age there was a significant effect of hyperoxia exposure on creatinine clearance (*P*_Group_=0.02), with particularly low levels observed in female NH and H groups (*P* < 0.05 compared to female controls). At 11 months of age, creatinine clearance (*P* = 0.005) was significantly lower and FENa significantly higher (*P* = 0.01) in females compared to males; there were no differences between groups or sexes in any other functional parameter.

**Figure 3:**
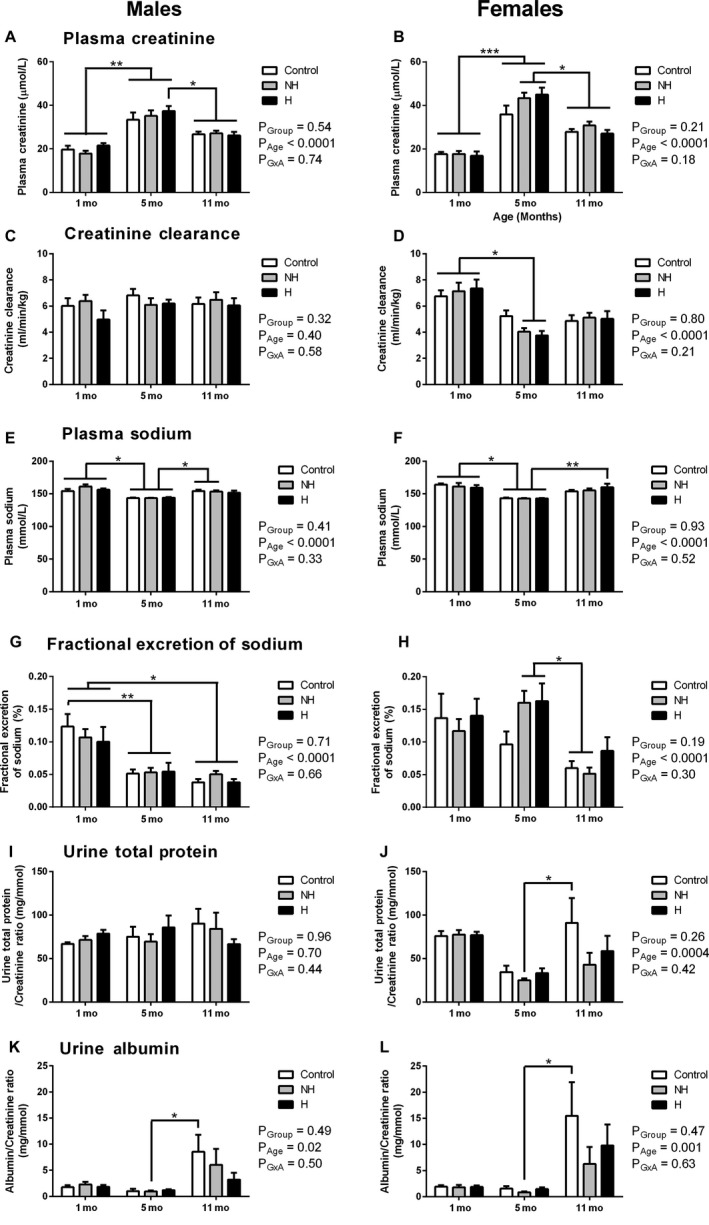
Renal function. Renal function parameters in male and female rats at 1, 5, and 11 months of age. Plasma creatinine (A, males; B, females), creatinine clearance (C, males; D, females), plasma sodium (E, males; F, females), fractional excretion of sodium (G, males; H, females), urine total protein (I, males; J, females), and urine albumin (K, males; L, females). Analysis by two‐way analysis of variance (ANOVA), with the factors group, age, and their interaction (*P*_G × A_). *P <* *0.05, **P <* *0.01, ***P <* *0.0001 between groups as indicated, according to Bonferroni post hoc analysis.

Changes in renal function with aging are shown in Figure 3. Plasma creatinine was significantly increased and plasma sodium significantly decreased in male and female animals at 5 months of age compared to both 1 and 11 months (Fig. 3). In males, the FENa significantly decreased with increasing age (Fig. 3G), but there was no impact of age on creatinine clearance (Fig. 3C). In females, FENa remained high at 5 months (particularly in NH and H females) before decreasing at 11 months (Fig. 3H); this was concurrent with a low creatinine clearance in NH and H females at 5 months (Fig. 3D). Urine total protein levels remained constant across the three ages in males, whereas in females it tended to decrease at 5 months. Urine albumin significantly increased at 11 months in both males and females (Fig. 3K and L).

### Renal corpuscle size

**Figure 4:**
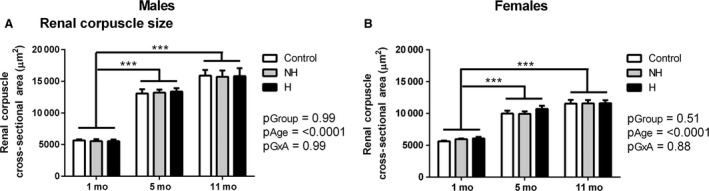
Renal corpuscle size. Renal corpuscle cross‐sectional area in male (A) and female (B) rats at 1, 5, and 11 months of age. Analysis by two‐way analysis of variance (ANOVA), with the factors group, age, and their interaction (*P*_G × A_). ***P <* *0.0001 between groups as indicated, according to Bonferroni post hoc analysis.

Average renal corpuscle cross‐sectional area significantly increased with increasing age in both male (Fig. 4A) and female (Fig. 4B) animals. At 5 and 11 months of age, renal corpuscle size was significantly larger in males compared to females (*P* < 0.0001), however there was no difference at 1 month. There was no impact of hyperoxia exposure on renal corpuscle size.

### Glomerulosclerosis and fibrosis

**Figure 5:**
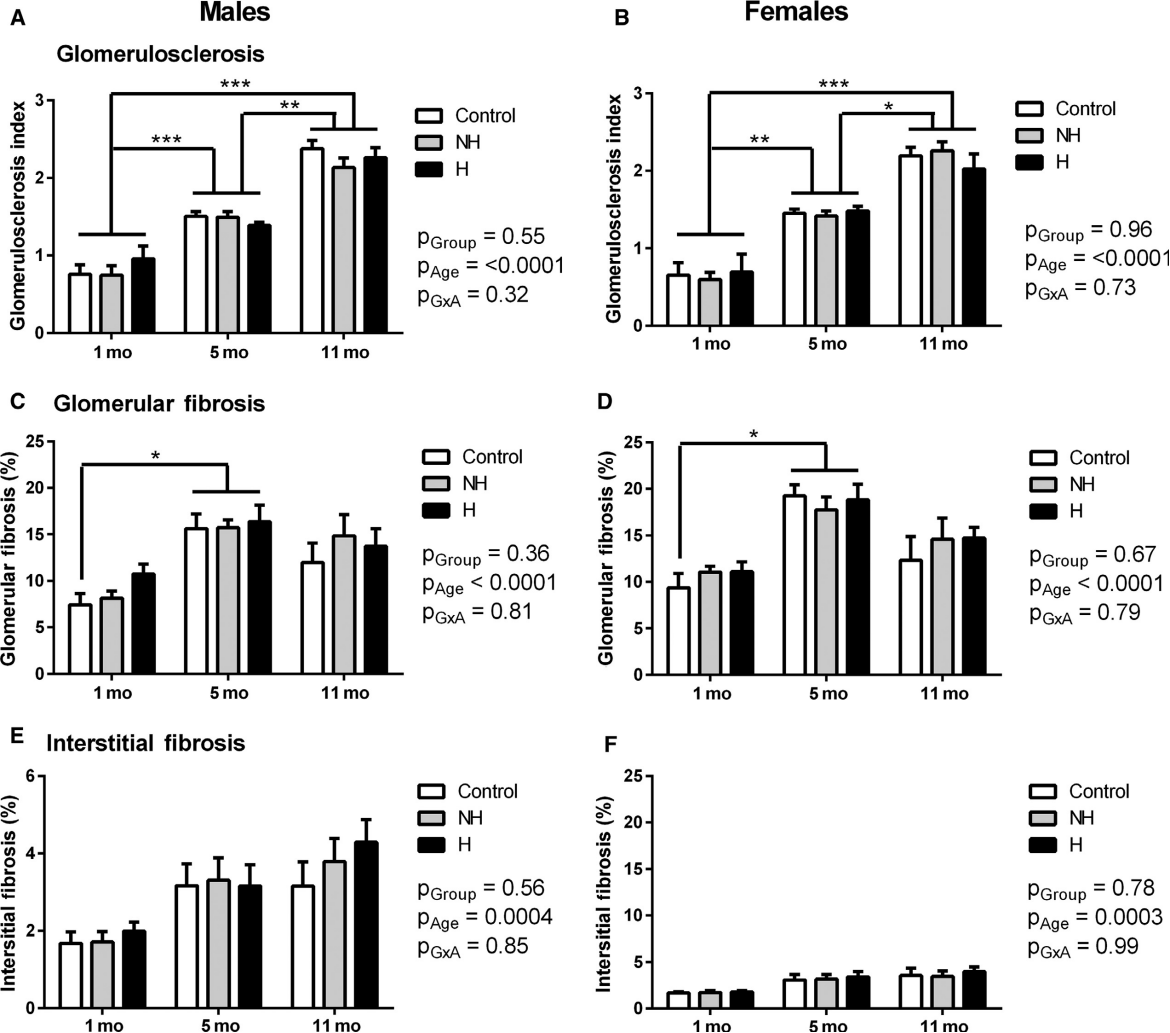
Renal pathology. Glomerulosclerosis index (A, males; B, females), glomerular fibrosis (C, males; D, females), and interstitial fibrosis (E, males; F, females) in animals at 1, 5, and 11 months of age. Analysis by two‐way analysis of variance (ANOVA), with the factors group, age, and their interaction (*P*_G × A_). *P < 0.05, **P < 0.01, ***P < 0.0001 between groups as indicated, according to Bonferroni post hoc analysis.

The severity of glomerulosclerosis (glomerulosclerosis index) significantly increased with increasing age in both males (Fig. 5A) and females (Fig. 5B). Similarly, the percentage of glomerular and interstitial fibrosis increased with increasing age (Fig. 5C–F). There was no significant difference in glomerulosclerosis between males and females at any age; females had significantly greater levels of glomerular fibrosis than males at 5 months of age (*P* = 0.03), but there was no difference between sexes at 1 or 11 months. Overall, there was no significant difference in glomerulosclerosis or fibrosis between groups.

### Glomerular crescents

**Figure 6:**
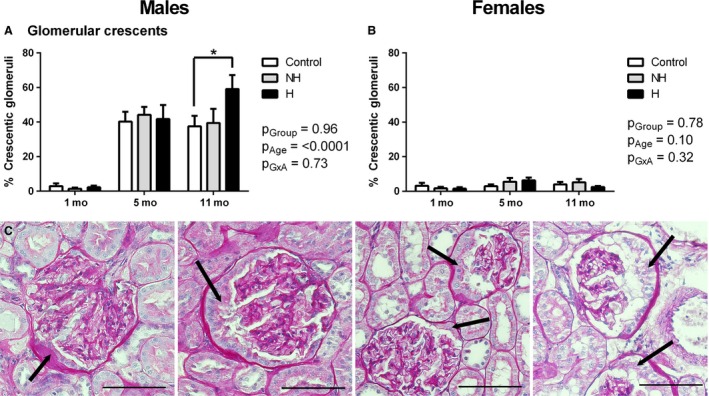
Glomerular crescents. Percentage of glomerular crescents (defined as an accumulation of extracellular material and/or cells in the Bowman's space, with no distinction made as to the composition or size of the crescents) in male (A) and female (B) rats at 1, 5, and 11 months of age. Analysis by two‐way ANOVA, with the factors group, age, and their interaction (*P*_G × A_). *P < 0.05 between groups as indicated, according to Bonferroni post hoc analysis. (C) Representative images of glomerular crescents (arrows) of differing morphology in PAS‐stained sections of kidney tissue from 11‐month‐old male H rats. Scale bars = 100 μm. ANOVA, analysis of variance; PAS, periodic acid Schiff.

The percentage of crescentic glomeruli (Fig. 6) was markedly greater in males at 5 and 11 months of age (*P* < 0.0001); in females, the percentage remained low throughout the 11 months. Importantly, the percentage of glomeruli with crescents was significantly increased in hyperoxia‐exposed (H) males at 11 months of age (59.1 ± 8.1%) compared to the normoxic controls (37.5 ± 6.2%) (Fig. 6A). The morphological appearance of the crescents is shown in Figure 6C.

### Macrophages

**Figure 7:**
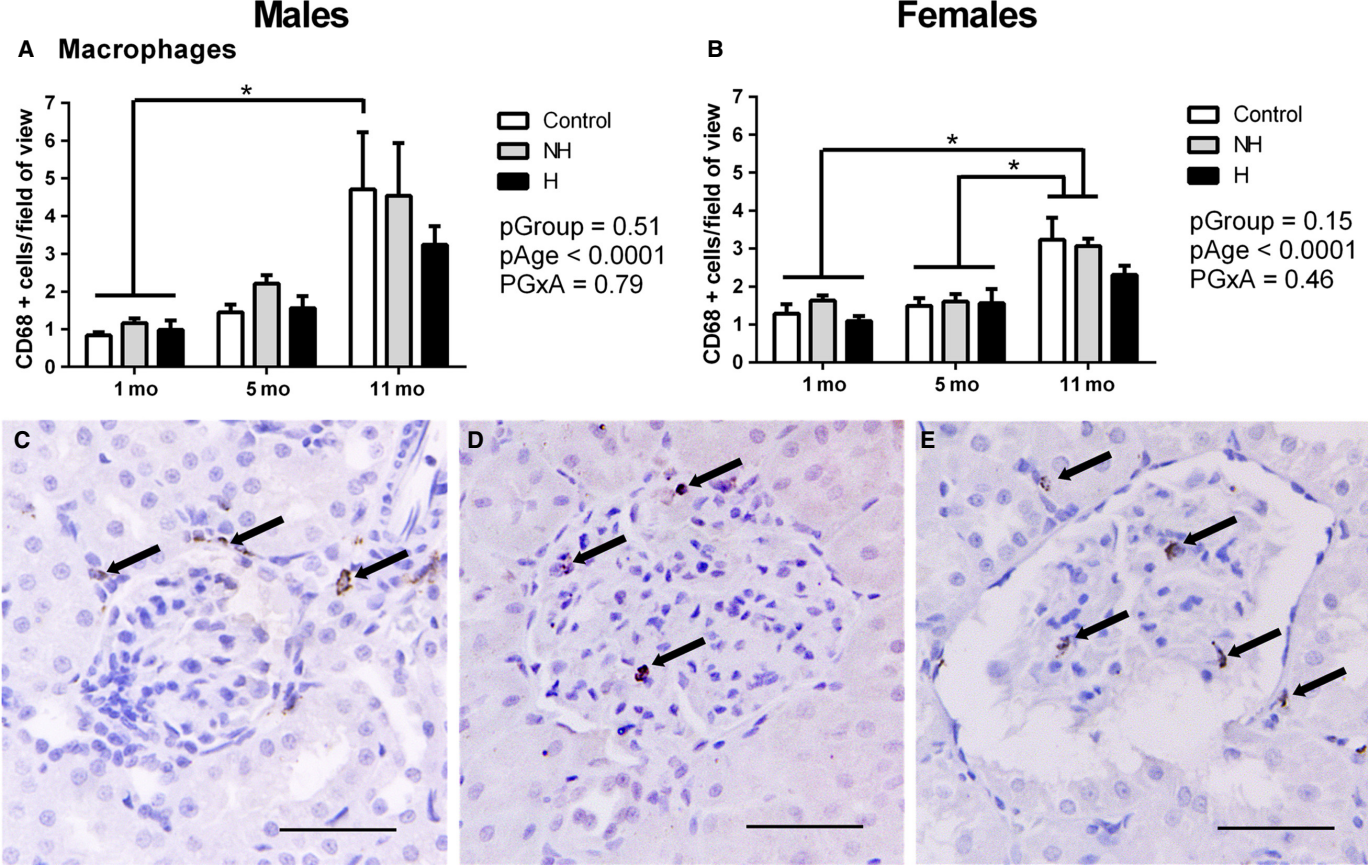
Macrophages. Average number of CD68+ cells (macrophages) per field of view in kidney tissue from male (A) and female (B) animals at 1, 5, and 11 months of age. Analysis by two‐way analysis of variance (ANOVA), with the factors group, age, and their interaction (*P*_G × A_). *P < 0.05 between groups as indicated, according to Bonferroni post hoc analysis. Representative images of CD68 immunohistochemistry in male H kidneys at 1 (C), 5 (D), and 11 (E) months of age. CD68+ cells are indicated by the brown staining and arrows. Scale bars = 50 μm.

The average number of macrophages in the renal tissue (Fig. 7) significantly increased with increasing age in male and female rats; macrophage number was greater in females than males at 1 month of age (*P* = 0.02), but there was no significant difference between sexes at 5 or 11 months. Overall, there was no significant impact of hyperoxia exposure on macrophage number. Macrophages were not commonly observed within glomerular crescents (Fig. 7E).

## Discussion

Since inception of the Developmental Origins hypothesis, there has been an emergence of evidence linking early life exposure to developmental insults to an increased risk of adult‐onset disease. In this study, we have shown that exposure to hyperoxia in the neonatal period (during active nephrogenesis) results in alterations in renal function and pathology in aging rats. These sex‐ and age‐specific findings included a significant reduction in creatinine clearance, and evidence of renal pathology (glomerular crescents). Exposure to high oxygen levels during nephrogenesis may therefore be an important factor underlying the observed renal dysfunction and pathology in human neonates and adults born preterm.

To date, there have been very few studies conducted focused on investigating the short‐ and long‐term impacts of hyperoxia exposure on the developing kidneys (Yzydorczyk et al. 2008; Popescu et al. 2013; Sutherland et al. 2013; Jiang et al. 2015). In a mouse model (65% O_2_ from P1 to P7), renal corpuscle size was found to be enlarged in animals at 8 weeks of age, suggestive of compensatory hypertrophy, but nephron number was not affected (as determined using the gold‐standard physical dissector/fractionator technique) (Sutherland et al. 2013). A recent study in rats (>95% O_2_ from P1 to P7, then 60% O_2_ from P7 to P21) also reported renal corpuscle enlargement, and increased levels of fibrosis in animals at 3 weeks of age (Jiang et al. 2015). In the neonatal rat model of early life exposure to hyperoxia used in the current study (80% O_2_ from P3 to P10), however, we have previously shown there is impaired nephrogenesis and increased apoptosis in the developing kidneys (Popescu et al. 2013); these findings were linked to a significant reduction in hypoxia‐inducible factor 1‐alpha (HIF‐1α) expression. In adulthood, animals exhibited high blood pressure along with a 25% reduction in nephron number (as determined using the acid‐maceration technique, which relies on the assumption that all renal corpuscles are equally resistant to acid) (Yzydorczyk et al. 2008). Given the dissonance of these findings, the mechanisms and consequences of oxygen exposure during nephrogenesis are far from understood; this study aimed to more comprehensively assess both renal function and morphology with aging.

### Impact of neonatal hyperoxia exposure on renal function

As expected, a number of age‐ and sex‐specific alterations in renal function were evident in this study. Rats at 5 months of age had the highest levels of plasma creatinine, likely due to greater muscle mass compared to the 1‐ and 11‐month‐old rats (Baxmann et al. 2008). Creatinine clearance did not alter throughout the 11 months in males; however, in females it significantly decreased with aging. In contrast, the FENa was significantly reduced at 5 and 11 months (indicative of increased sodium reabsorption) in males, whereas it remained high at 5 months in females before lowering by 11 months. Other studies of renal function in rats have widely variable results depending on strain, sex, age, cardiovascular health, and experimental protocol, making comparisons challenging (Goldstein et al. 1988). One factor which is common to the majority of studies is that males have a more accelerated age‐related functional decline than females which is linked to lower estrogen levels (Goldstein et al. 1988; Reckelhoff 1997; Black et al. 2015); this was not observed in the current study, although glomerular filtration rate (GFR) may be expected to remain stable until a later time point than was examined here (Goldstein et al. 1988; Reckelhoff 1997; Black et al. 2015). We did observe that urine albumin excretion was significantly increased in both males and females at 11 months of age (indicative of glomerular filtration barrier defects), this marked age‐related proteinuria is characteristic of laboratory rats (Goldstein et al. 1988).

Importantly, neonatal hyperoxia exposure was associated with an overall significant reduction in creatinine clearance when animals were assessed at 5 months of age. Females appeared to the most severely affected, however, and both NH and H females exhibited the highest FENa and plasma creatinine levels at this age. Given the number of factors potentially underlying a reduction in GFR (including low nephron number [Yzydorczyk et al. 2008] and altered renin–angiotensin system function [Bertagnolli et al. 2014, 2016]), the exact cause could not be determined in the current study. One likely possibility is tubule injury contributing to a reduction in creatinine excretion and/or sodium reabsorption, as pathological changes to the tubules have been previously reported in cases of oxidative stress in human preterm neonates (Vento et al. 2005; Perrone et al. 2007) and a rat model (Jiang et al. 2015). Therefore, it will be important for future research to focus on investigating the impact of neonatal hyperoxia on renal tubule development, function, and injury. Dehydration is another possibility as placement in the metabolic cages could have led to stress‐induced water avoidance (Farhan et al. 2014). It may be speculated that the stress response of the hyperoxia‐exposed animals is heightened compared to the controls, as has been documented in other developmental programming models and in individuals born growth restricted (Kett 2012). Only animals at 5 months (and not 1 or 11 months) were shown to have impaired creatinine clearance, which potentially relates to the high plasma creatinine levels (higher muscle mass and perhaps metabolic activity) at this age which may have been at the limit of their renal functional capacity to clear.

### Impact of neonatal hyperoxia exposure on renal pathology

Many sex‐ and age‐specific differences in renal morphology were observed in this study; in general, males had larger kidneys with a greater average renal corpuscle size than the smaller females throughout the 11 months. As expected to occur with aging (Goldstein et al. 1988), kidney weight, renal corpuscle size, and pathological findings (fibrosis and glomerulosclerosis) all increased in both males and females.

Although increased renal corpuscle size (suggestive of glomerular hypertrophy and hyperfiltration as compensation for a low nephron number, Puelles et al. 2011) has been observed in other rodent models of neonatal hyperoxia exposure (Sutherland et al. 2013; Jiang et al. 2015), there was no difference between groups in the current study. Previous studies also have had conflicting results regarding whether nephron number is actually significantly impacted by the hyperoxia exposure during nephrogenesis, either no difference (Popescu et al. 2013; Sutherland et al. 2013) or up to a 25% reduction (Yzydorczyk et al. 2008) has been reported. Given that different analytical methods, and animal models, were utilized for each study, the full impact of oxygen exposure on nephrogenesis is currently unknown. A limitation of this study is that nephron number was not assessed as we had done previously in other studies using the same experimental model (Yzydorczyk et al. 2008; Popescu et al. 2013); however, given that there were no other suggestive pathological changes, it is plausible that nephron number was not significantly affected.

Increased levels of renal (Jiang et al. 2015) and cardiac (Bertagnolli et al. 2014) fibrosis have been observed in recent studies of hyperoxia exposure, and increased glomerulosclerosis was hypothesized to occur due to the adult‐onset high blood pressure characteristic of this model (Yzydorczyk et al. 2008; Bertagnolli et al. 2014). Conversely, however, there were no significant changes in either parameter following neonatal hyperoxia exposure in aging rats of either sex. Given the link between oxidative stress and inflammation (Salzano et al. 2014; Sutherland et al. 2014), the number of macrophages (a strong correlate of renal disease progression, Duffield 2010) was also assessed, but no difference between groups was found. As the animals were assessed from 1 month of age, however, it is possible that inflammation would be present at an earlier time point during or immediately after the period of hyperoxia exposure.

One important finding was that of a marked increase in the percentage of glomeruli with crescents in the kidneys of hyperoxia‐exposed (H) males at 11 months of age. Interestingly, this high percentage of crescents was not observed in the NH rats (with maternal O_2_ exposure, and some impairment of kidney development observed in prior studies, Yzydorczyk et al. 2008; Popescu et al. 2013), and is therefore directly linked to neonatal exposure to high oxygen concentrations. Crescents are proposed to form following adhesion of the glomerular tuft to the Bowman's capsule, and are associated with proteinuria and inflammatory renal disease (Goldstein et al. 1988; Kriz and LeHir 2005; Cohen 2006). Crescent formation is common in male rats with aging (Goldstein et al. 1988), with males known to be more prone to renal disease than females (Goldstein et al. 1988; Reckelhoff 1997; Black et al. 2015); in the current study, the percentage of crescents rose in male rats from 5 months of age, whereas it remained very low in females throughout the 11 months. The mechanisms underlying the observed link between neonatal hyperoxia exposure and later‐life renal disease are unknown, but may be associated with direct glomerular injury from high blood pressure (Yzydorczyk et al. 2008; Bertagnolli et al. 2014), albuminuria (markedly increased at 11 months of age), or increased susceptibility due to impaired renal vascular development (Popescu et al. 2013; Sutherland et al. 2014) and/or to accelerated cellular senescence as a by‐product of the early oxidative stress (Sutherland et al. 2014). There was no direct link between the percentage of crescents and functional parameters in the hyperoxia‐exposed males, which supports the notion that they have arisen due to an increased susceptibility to injury, such as a potentially augmented inflammatory response to the normal age‐related increase in albuminuria. Potentially, the males were particularly susceptible given that studies have shown male mice exhibit significantly greater inflammatory and apoptotic responses to pulmonary hyperoxia, suffering more extensive lung injury, than females (Lingappan et al. 2013, 2015). The majority of crescents were observed to be cellular, with no/little macrophage infiltration (Fig. 7) or fibrosis (Fig. 6), and therefore likely to be at a relatively early stage of the disease process. Importantly, however, with such a large degree of glomeruli affected, it would be expected that these animals would be at significant risk of later nephron loss leading to eventual renal failure in later life. With aging also comes an increased risk of “second‐hit” insults to the kidneys (such as obesity and diabetes), which would accelerate disease progression in kidneys that are already structurally and functionally compromised (Nenov et al. 2000; Abitbol et al. 2009).

## Conclusions

Overall, we have shown that an early life exposure to high oxygen levels during renal development does have long‐term adverse consequences for renal function and pathology. There is still a dearth of information, however, regarding the mechanisms and full impact of hyperoxia on the kidneys, and future research effort is certainly required to understand the long‐term risks to the health of preterm infants.

## Conflict of Interest

None declared.
